# Resource use efficiency of indoor lettuce (*Lactuca sativa* L.) cultivation as affected by red:blue ratio provided by LED lighting

**DOI:** 10.1038/s41598-019-50783-z

**Published:** 2019-10-01

**Authors:** Giuseppina Pennisi, Francesco Orsini, Sonia Blasioli, Antonio Cellini, Andrea Crepaldi, Ilaria Braschi, Francesco Spinelli, Silvana Nicola, Juan A. Fernandez, Cecilia Stanghellini, Giorgio Gianquinto, Leo F. M. Marcelis

**Affiliations:** 1 DISTAL – Department of Agricultural and Food Sciences, ALMA MATER STUDIORUM – Bologna University, Bologna, Italy; 2 DISAFA-VEGMAP, Department of Agricultural, Forest and Food Sciences, University of Turin, Turin, Italy; 3 Departamento de Ingeniería Agronómica, E.T.S. Ingeniería Agronómica, Universidad Politécnica de Cartagena, Cartagena, Spain; 4 Horticulture & Product Physiology Group, Wageningen University, Wageningen, The Netherlands; 5 Flytech s.r.l., Belluno, Italy; 6 Wageningen UR Greenhouse Horticulture, Wageningen, The Netherlands

**Keywords:** Environmental impact, Light stress

## Abstract

LED lighting in indoor farming systems allows to modulate the spectrum to fit plant needs. Red (R) and blue (B) lights are often used, being highly active for photosynthesis. The effect of R and B spectral components on lettuce plant physiology and biochemistry and resource use efficiency were studied. Five red:blue (RB) ratios (0.5-1-2-3-4) supplied by LED and a fluorescent control (RB = 1) were tested in six experiments in controlled conditions (PPFD = 215 μmol m^−2^ s^−1^, daylength 16 h). LED lighting increased yield (1.6 folds) and energy use efficiency (2.8 folds) as compared with fluorescent lamps. Adoption of RB = 3 maximised yield (by 2 folds as compared with RB = 0.5), also increasing leaf chlorophyll and flavonoids concentrations and the uptake of nitrogen, phosphorus, potassium and magnesium. As the red portion of the spectrum increased, photosystem II quantum efficiency decreased but transpiration decreased more rapidly, resulting in increased water use efficiency up to RB = 3 (75 g FW L^−1^ H_2_O). The transpiration decrease was accompanied by lower stomatal conductance, which was associated to lower stomatal density, despite an increased stomatal size. Both energy and land surface use efficiency were highest at RB ≥ 3. We hereby suggest a RB ratio of 3 for sustainable indoor lettuce cultivation.

## Introduction

**Figure 1:**
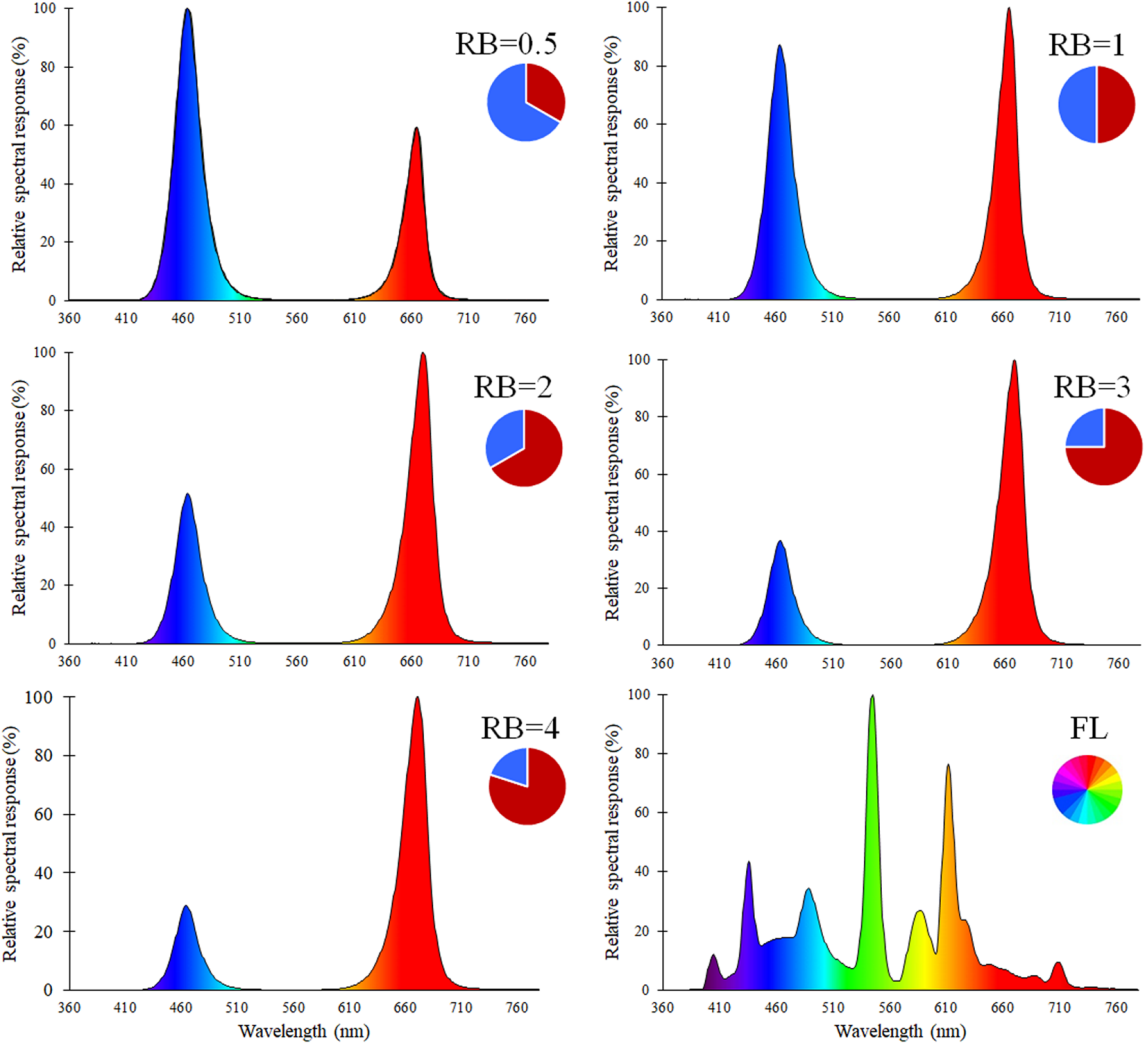
Light spectra used in the experiments.

In the coming decades, agricultural production may be constrained by limited availability of water^1^ and mineral nutrients^2^ as well as reduced availability of labour and accessibility to land and fertile soil^3^. Concurrently, worldwide increases in urban population are resulting in higher food needs^4^. Indoor vertical farms with full electric lighting have been suggested as a strategy to overcome land and resources scarcity by increasing water and nutrients use efficiency, thanks to the complete control of environmental factors and the limited exchanges with the external environment^5^. A simulation study with lettuce^6^ showed that vertical farms can increase land, water and nutrients use efficiency as compared to greenhouses located in Sweden, The Netherlands or the United Arab Emirates, although at the cost of elevated energy needs mainly associated with electric lighting. These achievements are related with the higher number of plants per unit land area thanks to the use of multiple growing layers^7^, but also to the possibility to recover water lost by transpiration and excess minerals and water draining from the growth media^5^. As for the quality features, the available possibilities for manipulating environmental conditions inside vertical farms were reported to allow for increased nutraceutical value (e.g., with reference to antioxidant content^8^) or decreased contamination risks (e.g., heavy metal deposition, pesticide application or nitrate accumulation in leaf tissues^7^). To date, however, the main constraints that limits large scale application of vertical farms are the high initial investments and the higher costs associated with energy consumption for illumination, cooling, heating, and dehumidification^6^. Nevertheless, as technology in LED application successfully evolves toward increasing the efficiency in converting electricity into light useful for photosynthesis (e.g. with values up to 2.6 μmol J^−1^, according to Radetsky^9^), a growing number of studies suggest that the overall efficiency of the system is highly dependent on the lighting strategy (e.g. spectral composition, intensity and photoperiod^10,11^). Accordingly, specific portions of the spectrum are considered to be major energy sources for photosynthesis^12^, given that the quantum yield curve of chlorophyll contains two peaks at the red (600–700 nm) and blue (400–500 nm) ranges. As a consequence, it is generally assumed that LED light use efficiency for indoor plant cultivation is highest with a combination of red and blue LEDs^13^. A major share of the available research on vertical farming systems has targeted the cultivation of lettuce (*Lactuca sativa* L.), due to its short growth cycle, the small plant size and the overall adaptability to intensive growing conditions^14^. The combined search for the terms *Lettuce* and (*plant factory* or *vertical farm* or *light emitting diode* or *indoor cultivation* or *indoor farm*) in SCOPUS® database resulted in more than 280 research papers in the last ten years, with more than 50 papers published on the topic only in 2018. Nevertheless, the great variability of the spectra used in currently available literature (e.g. only RB = 8 against fluorescent or monochromatic red LED in Yorio^15^; RB = 1 against monochromatic blue or monochromatic red in Johkan^16^, or RB = 3 against a mixture of red, green and white LEDs or a fluorescent light in Lin^12^) does not allow for identifying the optimal red:blue (RB) ratio in the light spectrum for lettuce cultivation. Indeed, what emerges is that the adaptation of the currently available plant growing technologies into indoor farming systems is a complex puzzle that requires a systematic approach, starting with the definition of the optimal light spectrum. Accordingly, the present study aimed at the identification of how variations in the RB ratio of the incident light (see spectra in Fig. 1) affect sustainability and resource use efficiency in indoor grown lettuce, by dissecting growth performance and related physiological and biochemical adaptations of the plant.

## Results and Discussion

### Lettuce response to changes in spectral RB ratio

**Figure 2:**
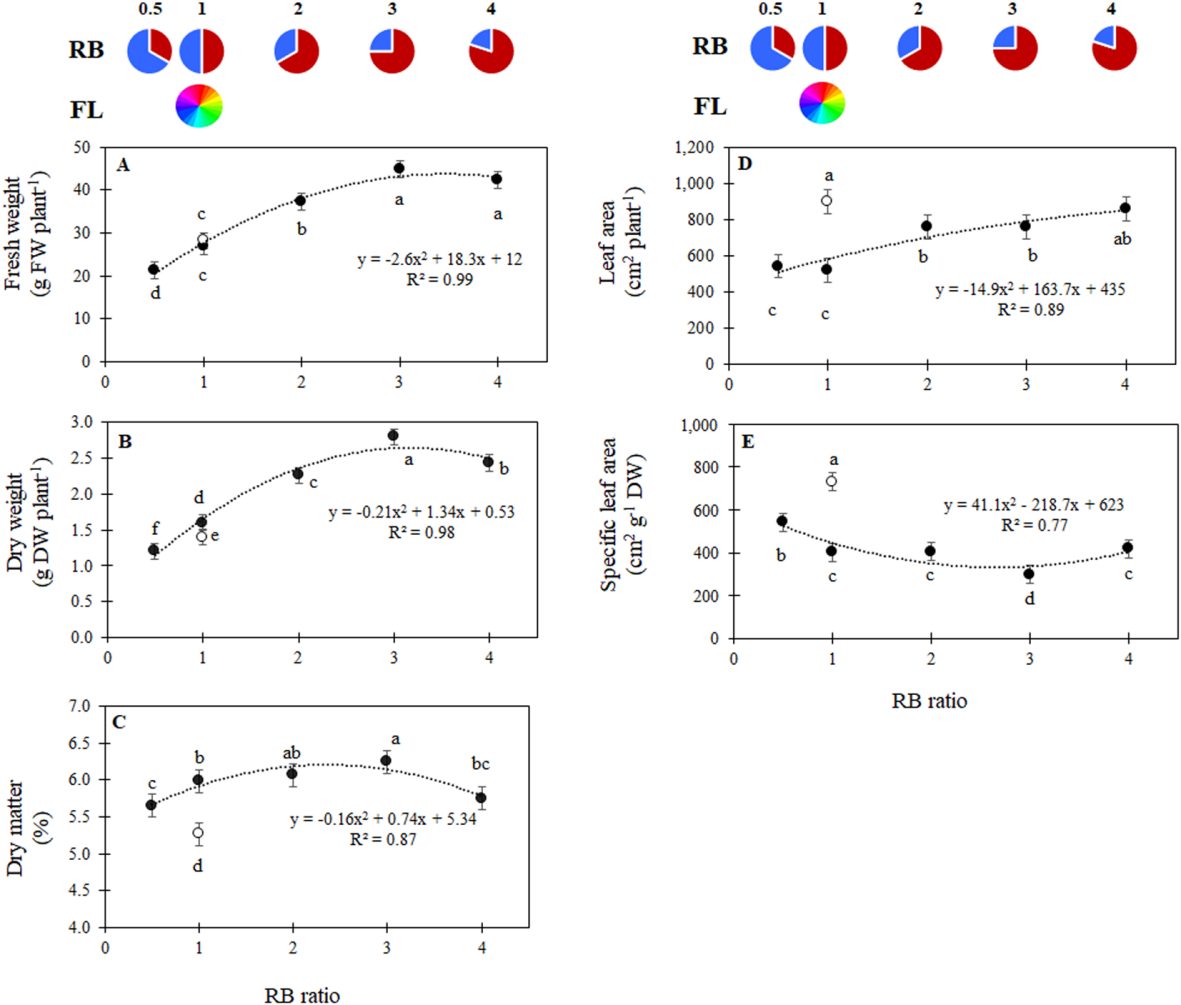
(**A**) Plant fresh weight, (**B**) plant dry weight, (**C**) dry matter content, (**D**) leaf area, and (**E**) specific leaf area (SLA) for lettuce plants grown under LED lights with different RB ratios (closed symbols) or under fluorescent light (open symbols). Mean values ± SE (45 replicate plants). Different letters indicate statistically significant differences at P ≤ 0.05.

Due to the absence of significant interaction between light treatment and experiment within the trials realised at Bologna University, mean values for each treatment from the five experiments (exp. 1, 2, 3, 4 and 5) are hereby presented and discussed. Fresh yield progressively increased from RB = 0.5 to RB = 3, whereas no further increase in yield was observed when RB exceeded 3 (Fig. 2A). No differences in yield were observed between FL and LED lights at similar RB value (RB = 1) (Fig. 2A). A similar yield response to RB ratio was also observed during exp. 6, where RB = 3 resulted in a yield increase by 34% as compared with RB ≤ 1 (average values from RB = 0.5 and RB = 1, data not shown). Previous evidence of the promotion of lettuce growth associated with mixed red and blue light was provided by Johkan^16^, where plants grown under monochromatic red light had lower yields when compared with plants grown under lights supplying RB = 1. On the other hand, monochromatic red was shown to promote shoot yield in both red and green lettuce, as compared with a number of red and blue combinations in another study^17^. Fresh yield was also increased when lettuce plants were grown under monochromatic red light as compared with mixed red and blue or a monochromatic blue light^18^, albeit at the expense of a lower plant dry matter content. It shall be noted, however, that the combination of red and blue light was beneficial for lettuce plant growth as compared with either monochromatic blue or monochromatic red light in another experiment^15^, overall suggesting that there is an optimum RB ratio. In the current study, the yield increase associated with the increasing red fraction of the spectrum only occurred up to RB = 3, and higher RB values did not result in additional lettuce yield (Fig. 2A). Similarly, plant dry weight increased by the increase of the red light component up to RB = 3, and it decreased at RB = 4 (Fig. 2B), suggesting an optimum response of dry weight to red and blue light ratios, as recently confirmed in greenhouse grown tomato undergoing supplementary LED lighting^19^. The increase in dry weight in response to additional red light was previously suggested by Snowden^20^ who reported the highest dry yield in lettuces grown under LED lights featuring RB ranging between 4 and 7 as compared with higher or lower RB values. Under fluorescent light, plant dry weight was significantly smaller than under the LED treatment supplying RB = 1 (Fig. 2B). The beneficial effects of LED lighting on dry weight accumulation were previously evidenced in studies where a control treatment with fluorescent lamps^16^ or a combined fluorescent + high pressure sodium lamp^17^ was included. From the same study^17^, it was also concluded that the combination of fluorescent and high pressure sodium light resulted in lower dry matter content in shoots of lettuce as compared to their growth under LED light supplying a mixture of red and blue light with an RB up to 3, whereas, higher RB ratios or a monochromatic red light reduced dry matter content. In our experiments, dry matter accumulation followed an optimum function, with the highest dry matter content associated with RB = 2 and RB = 3 (Fig. 2C). As compared to plants illuminated with a similar RB ratio provided by LED lamps, those grown under fluorescent lamps (FL) presented significantly lower dry matter content (Fig. 2C) due to lower plant dry weight (Fig. 2B) despite a statistically similar fresh weight (Fig. 2A). It was previously suggested that lettuce grown under fluorescent light compensates for the reduced photosynthetic efficiency with greater light interception by a larger leaf area^12^, as also observed in the current study, where FL presented the highest leaf area (Fig. 2D), with similar values being achieved only with LED light with RB = 4. The increased leaf area under FL light, but lower dry mass accumulation (Fig. 2B) resulted in higher specific leaf area (SLA) (Fig. 2E). The larger leaf area and higher SLA of lettuce grown under fluorescent lights instead of red + blue light was previously associated with puffiness (e.g. softer leaves) and a loose shoot structure of the former^12^, a response that was observed during our experiments.

Among LED treated plants, the highest leaf area was observed under RB ≥ 2 (Fig. 2D). Our results are consistent with the findings reported by Snowden^20^, who observed that the leaf area was reduced for lettuce grown under 200 μmol m^−2^ s^−1^ PPFD when the blue light fraction of the RB spectrum was increased. This dwarfing effect of blue light was previously observed for rice^21^, grape seedlings^22^ and strawberry^23^. On the other hand, among LED treated plants, the highest SLA was observed at the lowest RB ratio (RB = 0.5; Fig. 2E), mainly due to the constant decrease of dry weight in response to decreased RB values (Fig. 2B).

**Figure 3:**
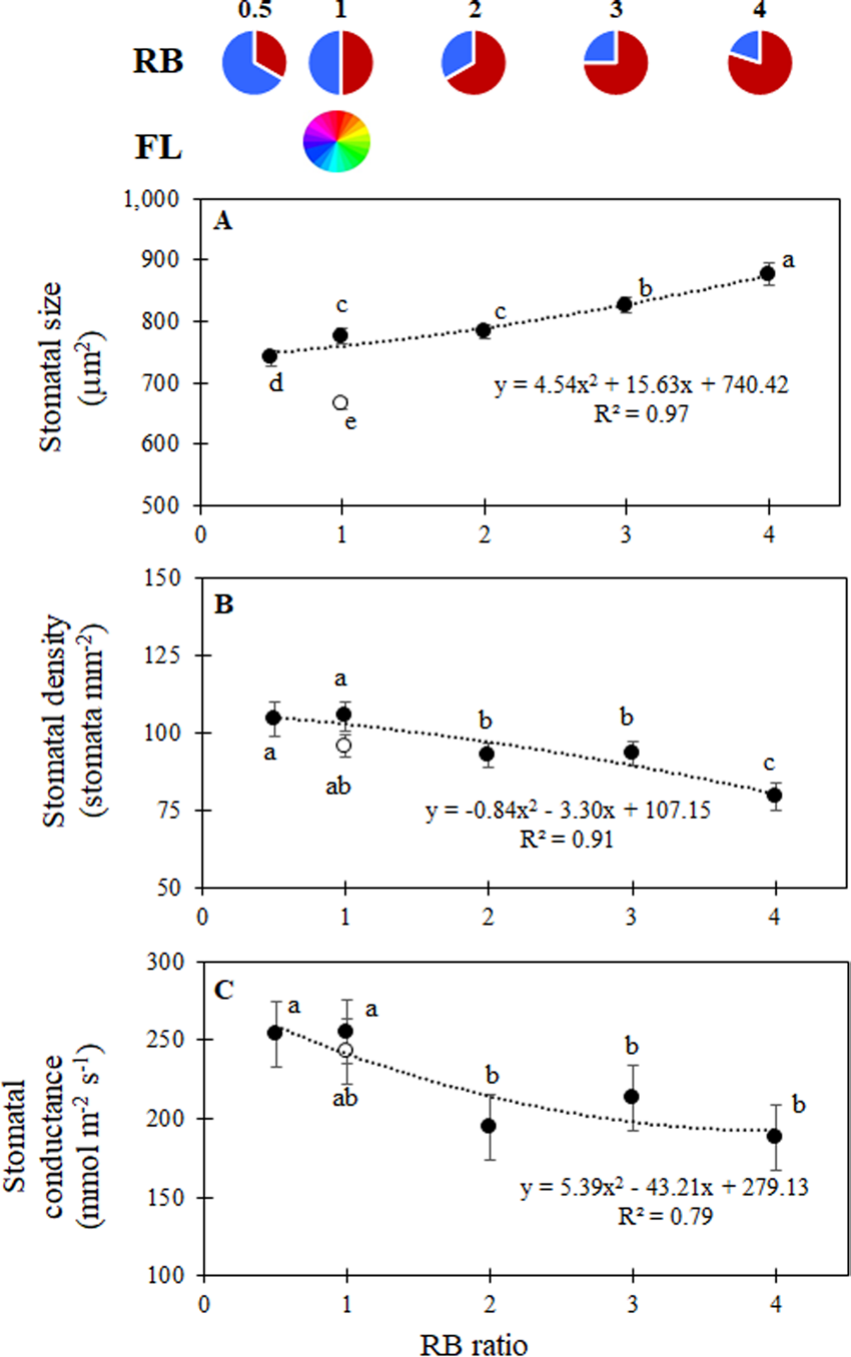
(**A**) Abaxial stomatal size, (**B**), abaxial stomatal density and (**C**) stomatal conductance for lettuce plants grown under LED lights with different RB ratios (closed symbols) or under fluorescent light (open symbols). Mean values ± SE (45 replicate plants). Different letters indicate statistically significant differences at P ≤ 0.05.

Other than leaf area, blue light may also influence leaf morphology, altering stomatal density and size, for instance in grapes^22^. For our study, stomatal size was generally increased by LED lights as compared with FL, and a progressive increase in size was also associated with the increased fraction of red light in the spectrum (Fig. 3A). On the other hand, stomatal density (Fig. 3B) and conductance (Fig. 3C) were shown to decrease when light with RB ≥ 2 was supplied. In a previous study, Wang^24^ reported that stomatal density and stomatal conductance decreased in lettuce leaves in response to increased RB ratios. Similarly, increases in the red light fraction were associated with a concurrent decrease in stomatal conductance in cucumber^25^. The role played by blue light in increasing stomatal conductance is a well-documented phenomenon^26^, which is due to the fact that stomatal guard cells open by the blue light receptors phototropins^27^. Increase in plant photosynthetic net assimilation associated with augmented blue light was also previously observed in lettuce plants grown under 500 μmol m^−2^ s^−1^ PPFD, whereas no significant differences in photosynthetic efficiency appeared when a number of red and blue spectra were compared at 200 μmol m^−2^ s^−1^ PPFD^20^. In our study, the plant PSII quantum efficiency (Fq’/Fm’, measured during exp. 6) decreased with increasing RB ratio; Fq’/Fm’ was 0.52, 0.51, 0.50, 0.50 and 0.48, respectively for RB values of 0.5, 1, 2, 3 and 4 (data not shown). In a previous study on cucumber^25^, the variations in the RB ratio presented a significant though limited effect on Fq’/Fm’, which decreased when the red portion was increased from 50% to 85%, 93% and 100%. A decrease in chlorophyll fluorescence was also observed in lettuce plants grown under prevailing red light as compared with increasing blue spectral fractions^17^.

**Figure 4:**
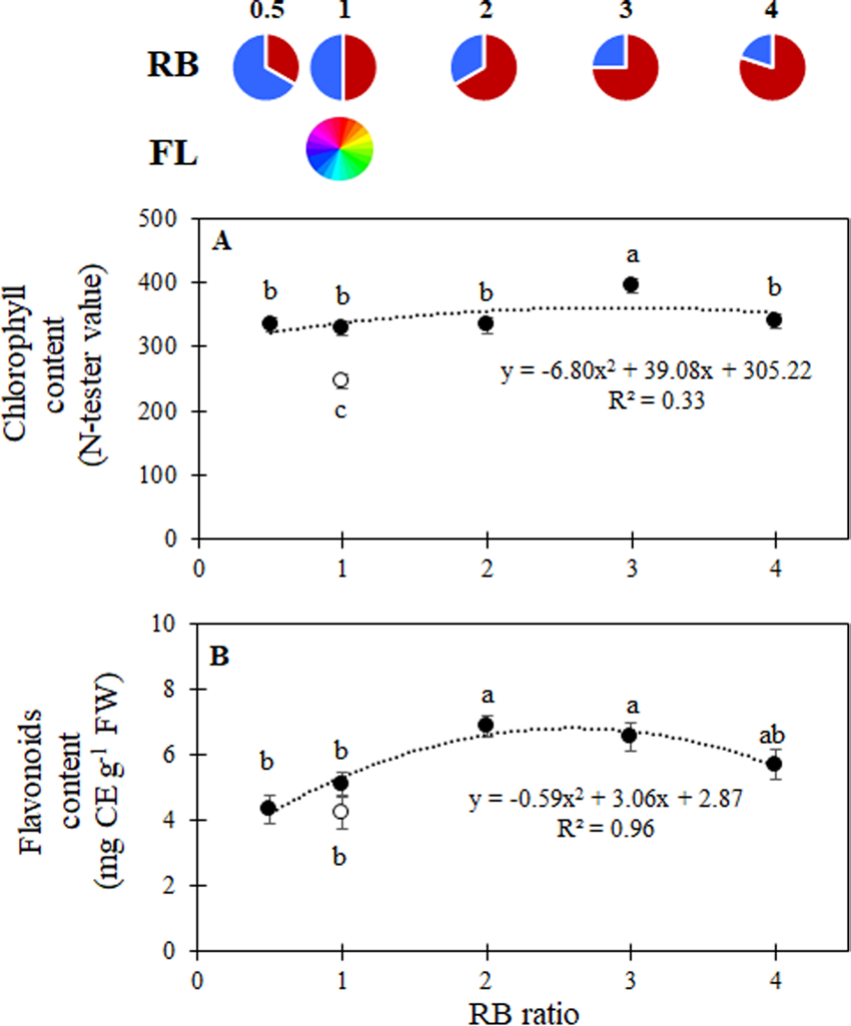
(**A**) Chlorophyll content and (**B**) flavonoids content for lettuce plants grown under LED lights with different RB ratios (closed symbols) or under fluorescent light (open symbols). Mean values ± SE (45 replicate plants for chlorophyll content, 27 replicate plants for flavonoids content). Different letters indicate statistically significant differences at P ≤ 0.05.

The chlorophyll concentration was higher in LED treated plants as compared with FL (Fig. 4A). Moreover, the highest chlorophyll content was found for RB = 3, whereas no differences were detected among other LED treated plants (Fig. 4A). During previous experiments, monochromatic red was repeatedly shown to lower chlorophyll values as compared with monochromatic blue or a red + blue light (RB = 1)^16,28^. Similarly, the highest chlorophyll content was also found when different RB ratios were provided, as compared with monochromatic red radiation^17^.

Flavonoids content was highest for RB ≥ 2, with the lowest values found for RB ≤ 1 (Fig. 4B). The increase in flavonoids is particularly interesting for their well-known nutraceutical quality and impact on dietary intake of antioxidant compounds^29^. A rise in flavonoids content has been observed in several plants in response to exposure to UV radiation^30^. In the absence of UV radiation, phenylalanine ammonia-lyase, which is a crucial enzyme in the phenylpropanoid pathway leading to flavonoid biosynthesis, has a significantly reduced activity^31^. However, increases in flavonoids were also observed in the presence of high irradiation in the absence of UV^32^. Red light was previously reported to improve antioxidant capacity in lettuce^33^. Furthermore, it was also shown that flavonoids were synthesised as photo-protectants in light-stressed lettuce plants^34^, supporting that, under prevailing red light, plants sacrifice photosynthetic efficiency^25^, while promoting antioxidant biosynthesis^11,33^. On the other hand, several reports found an increase in antioxidant compounds when additional blue light was supplied in lettuce^17^ as compared with monochromatic red light, suggesting an optimum response function, which in the current study resulted in maximum flavonoids accumulation for RB ≥ 2.

Nutrients uptake from the nutrient solution was generally affected by the light spectral properties (Table 1). The use of RB = 3 resulted in the greatest uptake of N, P, K and Mg. On the other hand, Ca uptake was not affected by the light spectrum supplied, while the uptake of Fe was highest for RB = 2 and RB = 4. Leaf concentrations of N, P and Fe were not affected by light treatment. K concentration was lowest under FL light. The treatments RB = 3 and FL resulted in the lowest Ca concentrations, whereas the Mg concentration in leaves was reduced under RB ≥ 2 (Table 1). Only a few preliminary studies on the effects of light spectrum on mineral accumulation in lettuce plants were performed in the past^28,35^, although the adopted experimental designs (without RB spectral gradients or the concurrent presence of other radiative components including green and far red) limit so far the possibility to form conclusions on which RB ratio would provide optimal nutrient uptake. Amoozgar^36^ reported monochromatic red light to increase leaf concentration of K, P and Fe in lettuce, while a mixed RB radiation enhanced N and Mg concentration in leaf tissues. However, Clavijo-Herrera^37^ demonstrated that N accumulation in lettuce leaves did not vary in response to changes in the RB ratio of the incident light, as was also shown for our experiments. Some indications on nutrients concentration in shoot tissues are available for other plant species, with studies addressing the role of red and blue radiation in P, K, Ca, Mg and Fe concentrations in broccoli microgreens^38^. In our previous studies^11^ we shed some light on how RB ratio affects mineral composition of basil plants, overall resembling the hereby shown results on lettuce, with RB = 3 resulting in greatest uptake of N, P and Mg. In the same study^11^, the highest concentrations of N, P, Ca and Fe were associated with RB = 3. From our results on lettuce, it appears that these nutrients are removed from the nutrient solution as a consequence of the increased plant biomass under RB = 3, while in leaf tissues the same light treatment only increased the concentration of K. This was corroborated by regression analysis of the nutrient uptake as a function of the dry weight showing a significant positive correlation (P < 0.05) for N (P = 0.000000), K (P = 0.000000) and Ca (P = 0.008879) (n = 108, df = 1,106, data not shown).

**Table 1:** Nutrient uptake, leaf concentration and nutrient use efficiency of selected mineral elements for lettuce grown under fluorescent light (FL) or LED lights with varying RB ratio (RB = 0.5, RB = 1, RB = 2, RB = 3 and RB = 4). Mean values based on 27 replicate plants. Different letters indicate statistically significant differences at P ≤ 0.05 (*), 0.01 (**) and 0.001 (***). ns = not significant differences.

	N						P						K					
	Uptake		Concentration		NUE_N_		Uptake		Concentration		NUE_P_		Uptake		Concentration		NUE_K_	
	%		(mg g^−1^ FW)		(g FW mg^−1^ N)		%		(mg g^−1^ FW)		(g FW mg^−1^ P)		%		(mg g^−1^ FW)		(g FW mg^−1^ K)	
RB = 0.5	36	c	3.17		0.32		37	bc	0.36		2.82		46	b	1.54	b	0.65	b
RB = 1	36	c	3.12		0.32		36	c	0.37		2.83		47	b	1.72	a	0.58	d
RB = 2	45	b	3.13		0.32		38	bc	0.38		2.68		57	ab	1.67	ab	0.60	cd
RB = 3	63	a	3.16		0.32		46	a	0.38		2.77		63	a	1.73	a	0.58	d
RB = 4	49	b	2.88		0.35		39	bc	0.34		3.01		59	ab	1.62	b	0.62	c
FL	41	bc	2.90		0.35		41	b	0.35		2.91		53	b	1.46	c	0.69	a
	***		ns		ns		***		ns		ns		***		**		**	
	**Ca**						**Mg**						**Fe**					
	**Uptake**		**Concentration**		**NUE**_**Ca**_		**Uptake**		**Concentration**		**NUE**_**Mg**_		**Uptake**		**Concentration**		**NUE**_**Fe**_	
	**%**		**(mg g** ^**−1**^ **FW)**		**(g FW mg** ^**−1**^ **Ca)**		**%**		**(mg g** ^**−1**^ **FW)**		**(g FW mg** ^**−1**^ **Mg)**		**%**		**(μg g** ^**−1**^ **FW)**		**(g FW mg** ^**−1**^ **Fe)**	
RB = 0.5	44		0.51	ab	1.98	b	25	b	0.54	ab	1.90	c	92	b	5.71		190.00	
RB = 1	43		0.53	a	1.93	b	26	b	0.56	a	1.88	c	90	bc	4.89		214.34	
RB = 2	47		0.52	a	1.95	b	28	b	0.48	b	2.15	bc	94	a	4.40		230.80	
RB = 3	50		0.46	b	2.15	b	32	a	0.38	c	2.67	a	92	b	5.16		209.81	
RB = 4	48		0.47	ab	2.11	b	27	b	0.45	bc	2.25	b	94	a	3.51		291.75	
FL	46		0.41	b	2.50	a	29	ab	0.42	bc	2.44	ab	89	c	4.87		245.30	
	ns		*		**		**		**		**		***		ns		ns	

### Resource use efficiency in response to RB ratio

**Figure 5:**
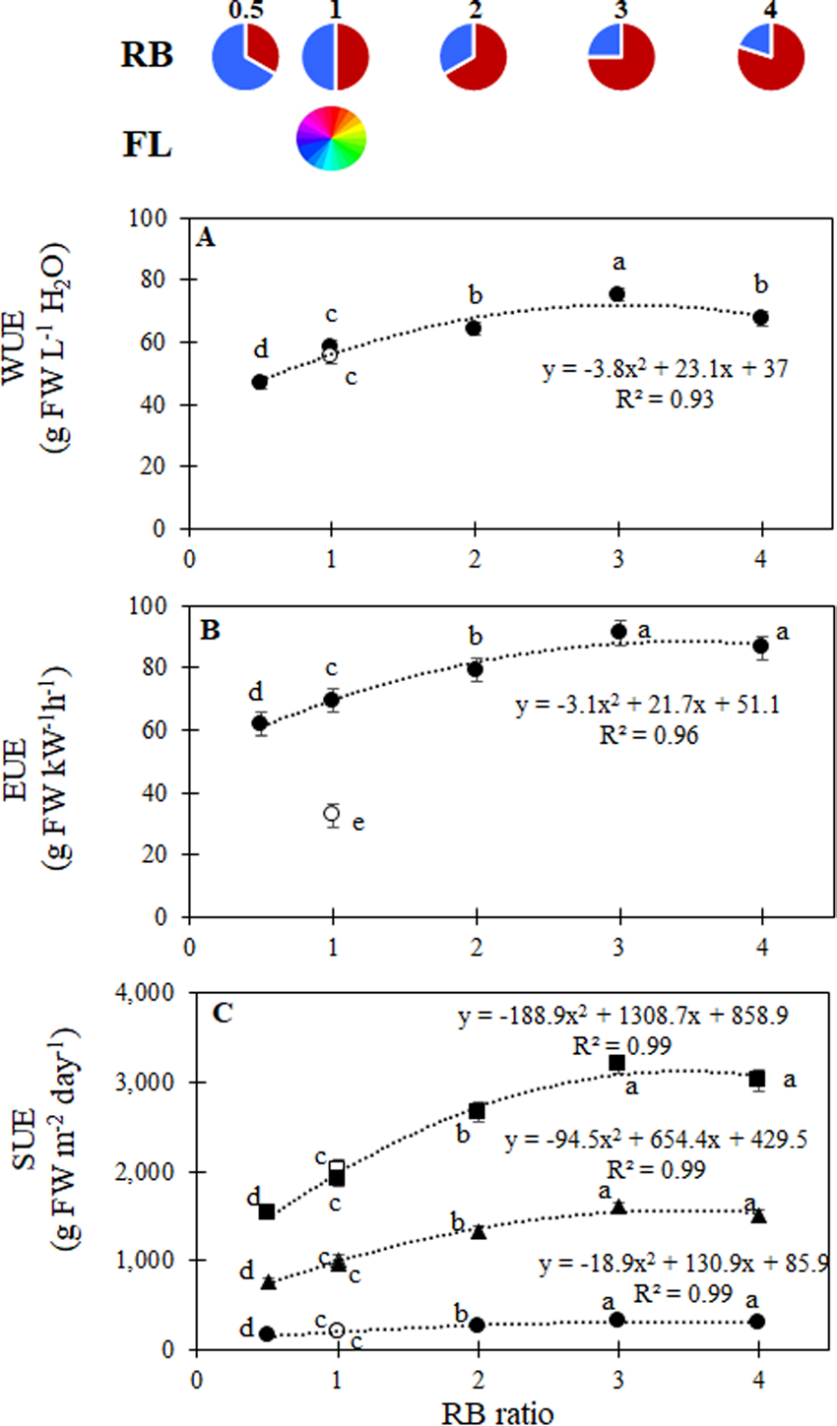
(**A**) Water use efficiency (WUE), (**B**) energy use efficiency (EUE), and (**C**) land surface use efficiency (SUE) for lettuce plants grown under LED lights with different RB ratios (closed symbols) or under fluorescent light (open symbols). For SUE chart (**C**), different shapes represent 1 layer (circles), 5 layers (triangles) or 10 layers (squares). Mean values ± SE (45 replicate plants). Different letters indicate statistically significant differences at P ≤ 0.05.

Our results show that plant water use was affected by light treatment, with average values of 0.61 ± 0.02 L plant^−1^ in plants grown under RB ≥ 3, 0.58 ± 0.02 L plant^−1^ in RB = 2, 0.52 ± 0.03 L plant^−1^ in FL and 0.46 ± 0.01 L plant^−1^ in RB ≤ 1. As a consequence of the observed variations in plant growth, WUE progressively increased from RB = 0.5 (47 g FW L^−1^ H_2_O) to RB = 3 (75 g FW L^−1^ H_2_O), while it reduced at RB = 4 (68 g FW L^−1^ H_2_O) (Fig. 5A). The inverse relationship between stomatal conductance and water use efficiency is a well-documented phenomenon^11^, which has been also studied in indoor vertical farm environments^13^. Accordingly, the increased biomass production (Fig. 2A) associated with a tighter control of leaf transpiration (Fig. 3B,C) for RB = 3 resulted in the highest WUE^39^ (Fig. 5A). Among the benefits associated with indoor plant cultivation with hydroponics and artificial lighting, reduced water requirements is a main driver for promoting vertical farming systems, with water use efficiency values in some cases up to 30–50 folds higher than those observed in open-field or greenhouse cultivation^40^. When it comes to lettuce cultivation, a previous comparative study^41^ among producers in Arizona (USA) reported WUE to range from 4 g FW L^−1^ H_2_O in conventional farms where lettuce is grown in soil, up to 50 g FW L^−1^ H_2_O in hydroponic greenhouses. Similarly, in open field lettuce cultivation (Lokyver Valley, Australia), WUE was reported to range from 8 to 19 g FW L^−1^ H_2_O, depending on the irrigation method adopted^42^. Alternatively, lettuce production in vertical farms was suggested to reduce water needs by up to 95% as compared with Venlo type hydroponic greenhouses in The Netherlands in estimates based on a model study^6^.

Nutrient Use Efficiency for N, P and Fe (NUE, Table 1) was not affected by light treatment, as indicated by their concentrations in the plant tissue. The observed NUE values for N were similar to NUE values observed in previous studies^43^. Concurrently, plants grown under FL light had the highest NUE for K and Ca (Table 1). On the other hand, Mg was most efficiently used during both RB = 3 and FL treatments (Table 1). Variations in the concentrations of selected elements (including N, P, K, Ca, Mg and Fe) were preliminarily associated with varying spectra, including red, blue, green and far-red components^35^. Nevertheless, from the current experiment, it appears that the main driver of nutrient uptake is plant growth, rather than light induced physiological changes.

As compared with traditional greenhouse cultivation, another crucial element for ensuring the economic viability of indoor cultivation is the amount of energy needed to sustain artificial lighting^13^. In addition to the observed differences in PPFD:electricity ratio between fluorescent (0.55 μmol J^−1^) and LED (0.98 to 1.40 μmol J^−1^) lights used in this study, the spectral properties of the incident radiation altered the energy use efficiency, resulting in varying lettuce biomass production under the different treatments (Fig. 5B). Light energy used was highest for FL treatment, as compared to the LED treatments, as a consequence of the lower PPFD:electricity ratio values observed in the former^11^. As a consequence, EUE values for all LED treatments were higher than the EUE for the FL treatment (33 g FW kW^−1^h^−1^). Changes in both light energy use and crop yield resulted in increasing energy use efficiency by 44% from RB = 0.5 to RB ≥ 3 (Fig. 5B). As a matter of fact, although the PPFD:electricity ratio is higher for blue compared to red emitting diodes, the increased yield compensated for the higher electricity power consumption, being maximised at RB ≥ 3 (Fig. 5B).

Viability of indoor vertical farms may also be related to the potential for land use saving, which assumes great relevance in densely populated urban environments with limited potential for developing traditional agriculture systems^13^. Potential for increasing the SUE in vertical farms was previously estimated to be 10- to 100-folds compared to traditional agricultural systems^40^. Lettuce productivity per land surface used in vertical farms was estimated in 12.5 to 25 folds higher than the productivity obtainable in a Venlo type greenhouse in The Netherlands, when a 5- or 10-layers vertical farms was used^6^. In the current study, the achievable yield per unit land surface devoted to cultivation resulted in a SUE being maximised when plants were grown under RB ≥ 3, reaching yields of approximately 1555 or 3110 g m^−2^ d^−1^, when 5 or 10 layers were cultivated, respectively (Fig. 5C). These SUE values become remarkable (10 to 20 times higher)^6^ when compared with data reported in the existing literature on agricultural yield of current production systems (e.g. 115 g m^−2^ d^−1^ in hydroponic greenhouses in Arizona)^41^.

## Conclusions

The adoption of red and blue LED lighting with RB = 3 maximised lettuce yield (45 g plant^−1^), resulting in a potential land surface use efficiency (SUE) of 3110 g m^−2^ d^−1^ using a ten layer vertical farming system. Efficient use of both water (WUE up to 75 g FW L^−1^) and energy (EUE up to 91 g FW kW^−1^h^−1^) were also ensured at RB = 3. When RB < 3 was used, photosynthetic quantum efficiency and stomatal conductance were higher (due to increased stomatal density despite a lower stomatal size), and lower chlorophyll and flavonoids concentrations were found in leaves. In order to expand the knowledge on vertical farming systems, future studies should address the inclusion of further spectral components (e.g. green and far-red light), the selection of species and cultivar most adapted and viable in vertical farms, and the determination of optimal light intensity and photoperiod management.

## Methods

### Plant material and growth conditions

Six separate experiments were carried out in growth chambers equipped with light insulated compartments at the Universities of Bologna (Italy) and Wageningen (The Netherlands). Lettuce plants “Gentilina” (*Lactuca sativa* cv. Rebelina, Gautier, Eyragues, France) were used, at a density of 100 plants m^−2^. Plants were grown under electric light (photosynthetic photon flux density - PPFD - on the top of the canopy of 215 ± 5.5 μmol m^−2^ s^−1^, and photoperiod of 16:8 h of day:night), with environmental conditions featuring constant air temperature of 24 ± 2 °C, 55–70% relative humidity and 450 ppm of atmospheric CO_2_. Further details on cultivation systems, water and nutrient management are provided in the following paragraphs. The set-up of the experiments was largely the same as for the basil experiments described by Pennisi^11^.

### Light treatments

LED lamps used in all experiments were provided by Flytech s.r.l. (Belluno, Italy) and featured hyper red (peak at 669 nm) and blue (peak at 465 nm) emitting diodes, whose relative light intensity were set using a digital control system provided by the lamp supplier. Spectral properties were determined using an illuminance spectrophotometer (CL-500A, Konica Minolta, Chiyoda, Tokyo, Japan). A constant PPFD of 215 ± 5 μmol m^−2^ s^−1^ over the plant canopy was measured using a QSO (Apogee instruments, Logan, UT, USA) PAR Photon Flux Sensor (with equal sensitivity to red and blue radiation), connected with a ProCheck handheld reader (Decagon Devices Inc., Pullman, WA, USA). Electric power consumption (W = J s^−1^) was measured using a multimeter (Fluke 189, Fluke Corporation, Everett, WA, USA). All instruments were periodically checked and calibrated by manufacturers and when indicated by the manufacturer guidelines calibration was also performed at the time of measurements. In order to define the lighting system’s efficacy for converting electricity into light, the PPFD:electricity ratio (μmol J^−1^) was estimated through flat-plane integration technique as the ratio of the incident PPFD (μmol m^−2^ s^−1^) at a set distance (40 cm, equal to the height of the lamp from the canopy in all experiments) and the light electric power consumption (LEPC, W m^−2^ equal to J m^−2^ s^−1^)^10^. Such determination was previously shown to have a high regression coefficient (P = 0.004) with the Photosynthetic Photon Efficacy (PPE, μmol J^−1^), based on an integrating sphere determination^44^. LED treatments were characterised by five red:blue (RB) photon flux ratios of 0.5 (RB = 0.5, LEPC: 154 W m^−2^, PPFD:electricity ratio: 1.40 μmol J^−1^), 1 (RB = 1, LEPC: 172 W m^−2^, PPFD:electricity ratio: 1.25 μmol J^−1^), 2 (RB = 2, LEPC: 210 W m^−2^, PPFD:electricity ratio: 1.02 μmol J^−1^), 3 (RB = 3, LEPC: 219 W m^−2^, PPFD:electricity ratio: 0.98 μmol J^−1^) and 4 (RB = 4, LEPC: 219 W m^−2^, PPFD:electricity ratio: 0.98 μmol J^−1^). RB ratio was calculated by comparing the area under the curve of the spectral regions in the red (600–700 nm) and the blue (400–500 nm). Further description of lamp specific features are reported in Pennisi^11^ while spectral images are included in Fig. 1.

### Experiments at bologna university

The research was conducted at the Department of Agricultural and Food Sciences of Bologna University (Bologna, Italy) and was replicated in five independent experiments. During each experiment, six separate compartments (each 0.64 m^2^ surface and 0.4 m^3^ volume) of a climate controlled growth chamber were used. Each compartment was sealed with light opaque walls, internally painted in white, and equipped with fans constantly replacing internal air (200 air exchanges per hour). Before each experiment, full randomisation of light treatments was operated. Seeds were germinated in a potting media composed by a mixture of peat and vermiculite (70:30 v:v), under fluorescent lamps (FL, TL-D 90 De Luxe 58 W, Philips, Eindhoven, The Netherlands, featuring RB = 1, PPFD: 215 μmol m^−2^ s^−1^; LEPC: 386 W m^−2^, PPFD:electricity ratio: 0.55 μmol J^−1^). Fourteen days after sowing (with plants presenting two true leaves), plantlets were removed from the substrate by gently washing roots and transplanted into individual hydroponic systems (jars of 1 L of volume each) resembling a deep water culture^11^ with roots constantly submerged into a nutrient solution (electrical conductivity, EC: 1.6 dS m^−1^, pH: 6.5; N-NO_3_^−^: 14 mM; N-NH_4_^+^: 4.4 mM; P: 1.0 mM; K: 5.0 mM; S: 2.0 mM; Ca: 1.2 mM; Mg: 5.2 mM; Fe: 17.9 μM, Cu: 2.0 μM, Zn: 3.8 μM, B: 11.6 μM, Mn: 18.2 μM, Mo: 0.5 μM). The solution was continuously aerated through air pumps (Airline 3, Haquoss, Turin, Italy), providing an air supply rate of 0.25 L min^−1^ jar^−1^ and was never substituted, nor replenished before harvest. The six light treatments (one in each compartment) started at transplanting and included a control with fluorescent light (FL, with RB ratio of 1, details above) and five different LED light treatments where the RB ratio ranged from 0.5 to 4 (RB = 0.5, RB = 1, RB = 2, RB = 3 and RB = 4). Each compartment contained 48 plants (6 rows x 8 columns) and measurements were taken on the central 9 plants. Final measurements were taken 14 Days After the start of the light Treatment – DAT – meaning 28 Days After Sowing - DAS, at which stage the plants reached commercial harvest for loose leaves.

### Experiment at wageningen university

An additional experiment was conducted at Wageningen University (The Netherlands) to assess the response of the Photosystem II (PSII) Quantum Efficiency (Fq’/Fm’) to the changes in light spectrum. The experiment was performed in a climate controlled growth chamber (same settings as for the experiments in Bologna) where five separated compartments (each 0.4 m^3^) were created. Plants were grown in 6 cm pots filled with peat and watered with a nutrient solution (EC: 2.0; pH: 6.0; N-NO_3_^−^: 12.4 mM; N-NH_4_^+^: 1.2 mM; P: 1.1 mM; K: 7.2 mM; S: 3.3 mM; Ca: 4.1 mM; Mg: 1.8 mM; Fe: 25 μM; Cu: 0.8 μM, Zn: 5 μM, B: 30 μM, Mn: 10 μM, Mo: 0.5 μM), supplied as ebb- and flow irrigation once a day, still keeping the plants well-watered at any time. Seedlings were kept under LED lamps (Greenpower LED production module Deep Red/White 120 generation 1, Philips, Eindhoven, The Netherlands, featuring RB = 10 and PPFD: 215 μmol m^−2^ s^−1^) until reaching the two true leaf stage (21 DAS). According to energy consumption and light emission data provided by the lamp manufacturer, LEPC of the lamps was 120 W m^−2^ and PPFD:electricity ratio was 1.78 μmol J^−1^. Starting from 21 DAS, plants were distributed in the light insulated compartments within the growth chamber and LED light treatments were imposed, with the same conditions used during the previous experiments (RB = 0.5, RB = 1, RB = 2, RB = 3 and RB = 4, using the same lamps used during the experiments in Bologna, Fig. 1). Measurements of fresh yield and PSII quantum efficiency were taken at 10 DAT (31 DAS).

### Analyses and determinations

#### Growth analysis and resource use efficiency

At harvest (14 DAT), leaf fresh weight per plant (g FW plant^−1^) was measured in all experiments and dry weight per plant was quantified after drying the plants at 60 °C for 72 h. Dry matter was calculated as the ratio between leaf dry and fresh weights and expressed as percent value. Plant leaf area was determined using a leaf area meter (LI-300, LI-COR, Lincoln, Nebraska, USA), and Specific Leaf Area (SLA) was calculated as the ratio between plant leaf area and dry weight.

Water use was individually quantified for each plant during exp. 1, 2, 3, 4 and 5 and Water Use Efficiency (WUE) was determined as the ratio between plant fresh weight and the volume of water used, and expressed as g FW L^−1^ H_2_O. Lighting Energy Use Efficiency (EUE) was determined as the ratio between the final leaf fresh weight and the lamps’ cumulated electricity consumption and expressed as g FW kW^−1^h^−1^. Crop Nutrient Use Efficiency (NUE) was calculated as the ratio between leaf fresh weight and the concentration of selected nutrients (N, P, K, Ca, Mg and Fe)^11^ analysed in leaf tissues as described below. Land Surface Use Efficiency (SUE) was determined by analysing the potential achievable yield per unit land surface (with plant density of 100 plants m^−2^) over a year (26 crop cycles, considering 14 days per cycle from transplanting to harvest, while transplanting to take place the day after harvest) in three scenarios, featuring a single layer or a plant factory with 5 or 10 layers^11^.

#### Stomatal size, density and conductance

Measurements of stomatal size and density were performed using a nail polish print of leaf abaxial sides. Imprints were taken (during exp. 1, 2, 3, 4 and 5) from the middle portion of the blade between the midrib and the leaf margin, on the fourth fully expanded leaf from five plants per treatment per experiment. Each imprint was placed on a microscope slide and covered with a cover slip. Image data was acquired at 100X magnification using a brightfield biological microscope (MT4300H, Meiji Techno, Saitama, Japan) equipped with a digital camera (UK1175-C QXGA color, ABS GmbH, Jena, Germany). From each imprint, five pictures were taken in different locations. Pictures were analysed using ImageJ software (version 1.48 v, NIH, USA). For each picture, stomata number was counted and stomata size was estimated by the average of the area of the rectangle encasing each stomata^45^. Measurements of stomatal conductance (mmol m^−2^ s^−1^) were performed on the third fully expanded leaf using a leaf porometer (AP4, Delta-T Devices, Cambridge, UK) at 14 DAT (28 DAS) during experiments 1, 2, 3, 4 and 5.

#### Chlorophyll fluorescence

Measurements of PSII quantum efficiency (Fq’/Fm’) was performed during experiment 6, using a PlantExplorer™ (PhenoVation B.V., Wageningen, NL), at 10 DAT (31 DAS). This parameter indicates the efficiency at which light absorbed by PSII is used for the reduction of the primary quinone acceptor (Q_A_). The instrument was equipped with the same lamps used during cultivations (supplying respectively RB = 0.5, RB = 1, RB = 2, RB = 3 and RB = 4, Fig. 1, Flytech s.r.l., Belluno, Italy), which were kept on before and during measurements. Measurements were performed during 1-second periods, imposing an intensive light flash of 3500 μmol m^−2^ s^−1^ by a monochromatic red LED source (peak 660 nm).

#### Leaf chlorophyll content

The concentration of chlorophyll in leaves was estimated during experiments 1, 2, 3, 4 and 5 at 14 DAT using a leaf chlorophyll meter (YARA N-Tester, Oslo, Norway) on the third fully expanded leaf. The tool provides a numeric three-digit dimensionless value that is commonly expressed as N-Tester value and was previously used for leaf chlorophyll estimation in lettuce^46^.

#### Biochemical determinations

During experiments 1, 2 and 3, leaf samples were collected at harvest (14 DAT), immersed in liquid N_2_ and kept at −80 °C for biochemical analysis or dried at 60 °C for 72 h in a ventilated oven for elemental analysis. Total flavonoids were determined on one gram of frozen samples of lettuce leaves by aluminium chloride colorimetric assay, following the methodology explained in Pennisi^11^. Concentrations of nitrogen (N), phosphorus (P), potassium (K), calcium (Ca), magnesium (Mg) and iron (Fe) were measured both in nutrient solutions (at transplanting stage and at harvest) and in lettuce leaves (at harvest). To determine P, K, Ca, Mg, and Fe, an inductively coupled plasma-optical emission spectrometer equipped with a plasma source and an optical detector with a charge-coupled device (SPECTRO Analytical Instruments GmbH & Co., Kleve, Germany) was used^47^. Total nitrogen was determined using the elemental analyser Shimadzu TNM-1 (Shimadzu, Kyoto, Japan) and following the methodology described by Lavrnić^47^. After determining minerals content in both fresh and final nutrient solutions at transplanting and harvest, respectively, mineral uptake from the nutrient solution was calculated as the percentage of nutrients removed by the plants as related to the initial amount.

### Statistical analysis

Measurements were performed on nine plants per light treatment. For exp. 1, 2, 3, 4 and 5, data were analysed by two-ways ANOVA (light spectrum x experiment) and the means were compared by Least Significance Difference (LSD), at the 5% significance level. Regression analysis was conducted on the correlation between mineral nutrient uptake and plant dry weight, at the 5% significance level. Results from exp. 6 (fresh yield and PSII quantum efficiency) were analysed using one-way ANOVA and the means were compared by Least Significance Difference (LSD), at the 5% significance level. For all statistical analyses, software used included Microsoft Excel® and the SPSS package.
